# *Euglena mutabilis* exists in a FAB consortium with microbes that enhance cadmium tolerance

**DOI:** 10.1007/s10123-023-00474-7

**Published:** 2024-01-03

**Authors:** Emma Kaszecki, Daniel Palberg, Mikaella Grant, Sarah Griffin, Chetan Dhanjal, Michael Capperauld, R. J. Neil Emery, Barry J. Saville

**Affiliations:** 1 Environmental and Life Science Graduate Program, Trent University, Peterborough, ON Canada; 2 Forensic Science Department, Trent University, Peterborough, ON Canada; 3 Department of Biomedical Engineering, Carnegie Mellon University, Pittsburgh, PA USA; 4 Department of Biology, Trent University, Peterborough, ON Canada

**Keywords:** Bioremediation, Biotechnology, Heavy metals, Algal symbiosis, Co-culture, Fungal–algal–bacterial (FAB)

## Abstract

**Background:**

Synthetic algal–fungal and algal–bacterial cultures have been investigated as a means to enhance the technological applications of the algae. This inclusion of other microbes has enhanced growth and improved stress tolerance of the algal culture. The goal of the current study was to investigate natural microbial consortia to gain an understanding of the occurrence and benefits of these associations in nature. The photosynthetic protist *Euglena mutabilis* is often found in association with other microbes in acidic environments with high heavy metal (HM) concentrations. This may suggest that microbial interactions are essential for the protist’s ability to tolerate these extreme environments. Our study assessed the Cd tolerance of a natural fungal–algal–bacterial (FAB) association whereby the algae is *E. mutabilis*.

**Results:**

This study provides the first assessment of antibiotic and antimycotic agents on an *E. mutabilis* culture. The results indicate that antibiotic and antimycotic applications significantly decreased the viability of *E. mutabilis* cells when they were also exposed to Cd. Similar antibiotic treatments of *E. gracilis* cultures had variable or non-significant impacts on Cd tolerance. *E. gracilis* also recovered better after pre-treatment with antibiotics and Cd than did *E. mutabilis*. The recoveries were assessed by heterotrophic growth without antibiotics or Cd. In contrast, both *Euglena* species displayed increased chlorophyll production upon Cd exposure. PacBio full-length amplicon sequencing and targeted Sanger sequencing identified the microbial species present in the *E. mutabilis* culture to be the fungus *Talaromyces* sp. and the bacterium *Acidiphilium acidophilum*.

**Conclusion:**

This study uncovers a possible fungal, algal, and bacterial relationship, what we refer to as a FAB consortium. The members of this consortium interact to enhance the response to Cd exposure. This results in a *E. mutabilis* culture that has a higher tolerance to Cd than the axenic *E. gracilis*. The description of this interaction provides a basis for explore the benefits of natural interactions. This will provide knowledge and direction for use when creating or maintaining FAB interactions for biotechnological purposes, including bioremediation.

**Supplementary Information:**

The online version contains supplementary material available at 10.1007/s10123-023-00474-7.

## Background

The association of algae with fungi is perhaps best known from lichen symbioses, but these alliances occur in a wider range of systems in which bacteria and fungi exchange key metabolites with algae (Cantin et al. 2009; Croft et al. 2005; Matthews et al. 2017) or strengthen their stress responses (Ramanan et al. 2016; Zan et al. 2019). Algae have been co-cultured with bacteria or fungi to enhance levels of algal biomass (Berthold et al. 2019; Fuentes et al. 2016) or the production of high-value products (Lutzu and Dunford 2018; Magdouli et al. 2016; Toyama et al. 2019). One natural association studied was that between the euglenoid *Euglena mutabilis* and a fungus, found in acid environments with high levels of heavy metals (Nakatsu and Hutchinson 1988). In the present investigation, potential associations between *E. mutabilis* and a fungus, as well as bacteria, are investigated to assess the contributions of the co-cultured species to cadmium (Cd) tolerance of the *Euglena*.

Euglenoids are a group of unicellular flagellates with a diverse ecological distribution (Ebenezer et al. 2022; Kostygov et al. 2021). They fill similar ecological niches as algae and have been investigated for applications in pharmaceuticals, cosmetics, biofuels, bioremediation, and foodstuffs (Khatiwada et al. 2020a; Li et al. 2021; Nakashima et al. 2018; Toyama et al. 2019; Yamamoto et al. 2018). *Euglena gracilis* is the model organism for this group, as it is readily cultured and has been widely studied and characterized. However, its genome sequence remains unannotated (Borst and Sabatini 2008; O’Neill et al. 2015), and knowledge of gene content has relied heavily on transcriptome analyses (Ebenezer et al. 2019, 2022). Insight regarding microbial interactions with *Euglena* in various environments has been gained by establishing synthetic associations in culture. Notably, *E. gracilis* is incapable of synthesizing vitamins B_1_ and B_12_ and must obtain these from exogenous sources (Lukáčová et al. 2022). When it is cultured with bacteria that can produce these vitamins, *Lysinibacillus boronitolerans* or *Pseudobacillus badius*, *E. gracilis* is able to grow for several generations without exogenous vitamin application (Lukáčová et al. 2022). *Euglena* biomass and compound production were also enhanced when grown with the growth-promoting bacteria *Emiticicia* sp. EG_3_ (Toyama et al. 2019) or with *Vibrio natriegens*, a bacterium capable of synthesizing the phytohormone indole-3-acetic acid (IAA) (Kim et al. 2019b). While studying synthetic associations is informative and has led to many interesting findings related to fungal–algal–bacterial (FAB) associations, the full potential of *Euglena* co-cultures and FABs would be better revealed by investigating naturally occurring interactions such as those between *E. mutabilis* and its inextricably associated partner organisms (Casiot et al. 2004; Sabater et al. 2003; Searles et al. 2001).

*E. mutabilis* is an extremophilic Euglenoid that is often found in toxic environments such as peat bogs, volcanic lakes, and acid mine drainage (AMD) (Casiot et al. 2004; Gross et al. 2002; Sittenfeld et al. 2002). In fact, its ability to grow in these environments, where few other organisms can grow, has led to it becoming a bioindicator for AMD (Ackil and Koldas 2006; Halter et al. 2012a, b; Valente and Gomes 2007). Acid drainage is naturally produced as water seeps through iron sulfide-aggregated rocks; however, its production is dramatically enhanced by the disposal of industrial mining waste, which creates acidic pools of toxic substances, including high concentrations of heavy metals (HMs). AMD thus poses severe human and environmental health concerns. When *E. mutabilis* grows in these environments, it often associates with other microorganisms to form biofilms (Brake et al. 2004; Nakatsu and Hutchinson 1988; Ňancucheo and Barrie Johnson 2012; Sittenfeld et al. 2002; Yanagawa et al. 2021). An isolate of *E. mutabilis* from an acidic pond in the Northwest Territories (Canada) was found to contain a yeast, later identified as *Cryptococcus* sp. (Nakatsu and Hutchinson 1988). The fungus was originally considered a contaminate, but the culture could not be cured, and the authors concluded this was a form of *E. mutabilis*–fungal mutualism (Nakatsu and Hutchinson 1988). The difficulty in obtaining axenic *E. mutabilis* cultures was also noted during subsequent isolation attempts from toxic environments (Nakatsu and Hutchinson 1988; Prigent et al. 2017). This led us to hypothesize that partner organisms augment the stress tolerance of *E. mutabilis* and aid its ability to survive in acidic, metal-polluted environments.

The objective of the presented research was to test this hypothesis by investigating the impact of antibiotic treatments on the growth and Cd tolerance of a natural *E. mutabilis*, fungal, and bacterial co-culture. A culture of *E. mutabilis* with associated fungal and bacterial organisms originally from an AMD site in Timmins, Ontario, Canada, was investigated. Extensive attempts to axenically separate fungi and bacteria from the *E. mutabilis* were not successful. Therefore, in the present study, an array of five antibiotics and two antimycotics were used in systematic attempts to suppress the growth of the partner organisms in the presence and absence of 100 μM CdCl_2_. Our findings indicate that there is a significant decrease in the number of viable *E. mutabilis* cells across all antibiotics and CdCl_2_ treatments compared to *E.* mutabilis co-cultures that are only exposed to CdCl_2_. This indicates that the associated microbes play a role in modulating the HM stress responses of *Euglena*. Suppressing bacterial and fungal growth also revealed the complexity of the interactions in this naturally occurring FAB association and supports the further investigation of natural interactions as model FABs for use in microbe technologies, including bioremediation.

## Methods

### *Culture selection and growth of E. mutabilis and E. gracilis*

Field samples of *E. mutabilis* were obtained from the Canadian Phycological Culture Centre (CPCC, University of Waterloo, Canada). The strain of *E. mutabilis* (CPCC 657) contained unidentified bacteria and fungi. The CPCC did not have an axenic culture of *E. mutabilis*, therefore an axenic culture of *E. gracilis* (CPCC 95) was obtained to act as an experimental control. Both organisms were grown autotrophically in 250-mL Pyrex Erlenmeyer flasks capped with foam stoppers and aluminum foil under standard aeration (100 RPM on a Thermo Fisher Scientific MaxQ 3000) while cycling light and temperature (16:8 LD cycle at 260 µmol s^−1^ m^−1^; 24℃ ± 0.5℃ in light and 18℃ ± 0.5℃ in dark) in a Conviron PCG20 environmental chamber (Olaveson and Nalewajko 2000). Stock cultures were grown in a modified acid medium (MAM), a defined inorganic medium (Olaveson and Stokes 1989), with modifications recommended by the CPCC and adjusted to a pH between 4.3 and 4.5 (Canadian Phycological Culture Centre Centre: Modified acid medium (MAM) 2017). Filter-sterilized F/2 vitamin mix was added after the medium was autoclaved.

### Antibiotic preparation

The five antibiotics and two antimycotic stock solutions were prepared with appropriate solvents. Kanamycin monosulfate (Bioshop Cat. No. KAN201) and tetracycline hydrochloride (Fisher Scientific Cat. No. A39246) were reconstituted in sterile MilliQ (13.4 MΩ·cm) water at a concentration of 1280 μg/mL. Rifampicin (Fisher Scientific Cat. No. 5573031GM), chloramphenicol (Fisher Scientific Cat. No. AAB2084122), and cycloheximide (Fisher Scientific Cat. No. AAJ6690103) were reconstituted in MeOH, 100% EtOH, and 95% EtOH, respectively, at a concentration of 1280 μg/mL (Holstege 2014; Schneck et al. 1971; Szulczwski and Eng 1975). A penicillin–streptomycin blend was purchased in solution (Fisher Scientific Cat. No. 15140122) and diluted using sterile MilliQ (13.4MΩ·cm) water to a concentration of 1280 units/L. Amphotericin B was also purchased as a solution (Fisher Scientific Cat. No. 15290026), and its concentration was not modified (250 mg/L). Antibiotics were stored at − 20℃ in sterile polypropylene test tubes (VWR Cat. No. CA60819-761) with parafilm around the lid. Most of the antimicrobial agents selected have a broad spectrum of impact. Their mechanisms of action include inhibition of protein synthesis (kanamycin, bactericidal; tetracycline, bacteriostatic; chloramphenicol, bacteriostatic; penicillin–streptomycin, bactericidal) (Garrett and Won 1973; Kapoor et al. 2017), inhibition of nucleic acid synthesis (rifampicin, bactericidal; cycloheximide, fungicidal) (Amin et al. 2004; Garrett and Won 1973), and disrupting the cell membrane permeability (amphotericin B, fungicidal) (Amin et al. 2004; Garrett and Won 1973).

### E. mutabilis and E. gracilis antibiotic treatments

Cell counts of *Euglena* from stock cultures of *E. mutabilis* were performed using a hemocytometer (Hausser Scientific). A 1-mL volume of 500,000 cells was aliquoted into 1.5 mL microfuge tubes, which were centrifuged (6000RCF for 5 min), and the supernatant was removed using a micropipette. The pelleted cells were inoculated into a well of a 12-well culture plate (VWR Cat. No. 10861–556) containing 1 mL of media per well.

Separate 12-well culture plates (VWR Cat. No. 10861–556) were prepared for each antibiotic and antimycotic in the format of a minimum inhibitory concentration assessment used by Weigand et al. (2008) (Fig. S1a). Culture plates contained a sterile control (900 μL of MAM and 100 μL of antibiotic solvent), growth control (900 μL of MAM, 100 μL of sterile MilliQ (13.4MΩ·cm) water, and 500,000 cells), and growth “check” (900 μL of MAM, 100 μL of alcohol, and an aliquot of cells) if an antibiotic was reconstituted in an alcohol. Wells that contained antibiotics were prepared using serial dilutions beginning with 1800 μL of MAM and 200 μL of an antibiotic or antimycotic in well A4. Upon completion of serial dilutions, each well contained a final volume of 1000 μL of media with antibiotic concentrations of 64, 32, 16, 8, 4, 2, 1, 0.5, and 0.25 µg/mL. Due to its stock concentration, modifications were made to the volume of media and antimycotic in culture plates containing amphotericin B; however, the final antibiotic concentrations were the same (Fig. S1c). Cells were added to each well in the culture plate, except for the sterile control. Culture plates were sealed with parafilm and stored in the Conviron PGC20 environmental chamber under the same temperature and light conditions as the stock cultures with aeration (120 RPM) for 72 h. This time was previously determined to be the maximum length for which amphotericin B and penicillin–streptomycin remained fully effective (Perlman 1979).

An identical method and analysis were used for *E. gracilis*. Each experiment was replicated 3 times.

### Euglena cell viability after antibiotic and antimycotic exposure

Seventy-two hours post-inoculation, 100 μL aliquots were taken from each well containing cells. Then 400μL of a 0.4% solution of Trypan Blue (Bioshop Cat. No. TRY477.100) in PBS (Bioshop, Cat. No. PBS404.100) was added to each aliquot. *Euglena’s* cell viability was assessed by visualizing differential uptake of the Trypan Blue stain, and cell counts were performed using a hemocytometer (Hausser Scientific). Each sample was counted twice per replicate, for a total of 6 counts per treatment. This was carried out on both *E. mutabilis* and *E. gracilis*.

### Assessment of chlorophyll content

A modification of the methanol (MeOH) extraction procedure developed by Warren (2008) was used to extract chlorophyll (Warren 2008). In the modified procedure, 500 μL aliquots from each well in the culture plates were added to 2 mL Eppendorf tubes containing 200 μL of glass beads (Sigma Aldrich Cat. No. G8772). The media was decanted after centrifugation at 4℃ (maximum speed for 2 min); 1 mL of chilled MeOH was added to the Eppendorf tubes, and samples were bead-beat at 30 Hz for 2 min (Retsch Mixer Mill MM 400). Tubes were then centrifuged at 4℃ (maximum speed for 2 min), and MeOH was aliquoted into a fresh 2-mL Eppendorf tube. This method was repeated so the final volume of extracted chlorophyll was 2 mL in MeOH; 200 μL of extract was added to each of three 96-well optic plates (ThermoFisher Scientific Cat. No. 165305) for triplicate readings on a BioTek Synergy HTX Multimode Reader at 652 and 665 nm wavelengths. Chlorophyll content was determined using the equations from Warren (2008), and chlorophyll content was normalized against the amount of chlorophyll in each *Euglena* cell.

### Growth recovery of cultures after antibiotic treatment

Recovery of *E. gracilis* and *E. mutabilis* cultures after growth in antibiotic and CdCl_2_ was assessed by plating on media without selection and allowing heterotrophic growth. Reasoner’s 2A (R2A) and potato dextrose agar (PDA) plates were prepared using a 1.4% (w/v) agar mixture. Solutions were autoclaved to sterilize, and then 15 mL of media was added to each plate (Greiner Bio-One Cat. No. 633181). In MilliQ (13.4MΩ·cm) water, 100 μL aliquots were taken from wells in the culture plates containing cells and diluted with 900 μL of sterile 0.9% NaCl; 50 μL of diluted sample was added to an agar plate, sealed with parafilm, and incubated in darkness at 24℃ for 7 days. Cultures treated with antibiotics were plated on R2A, while cultures treated with antimycotics were plated on PDA. The number of *Euglena*, fungal, and bacterial colony-forming units (CFUs) were noted every 24 h throughout the 7-day incubation period.

### Cadmium tolerance of E. mutabilis and E. gracilis with antibiotics

Kanamycin, rifampicin, chloramphenicol, tetracycline hydrochloride, penicillin–streptomycin, and amphotericin B at concentrations of 64, 32, 16, 8, 4, and 2 µg/mL were used to assess the impact of antibiotics and antimycotics on the CdCl_2_ tolerance of *E. mutabilis* and *E. gracilis*. Cultures treated with cycloheximide at concentrations higher than 16 μg/mL did not contain viable cells, and therefore this antimycotic was not used in these CdCl_2_ trials.

The inoculation of 12-well plates was carried out in the same manner as indicated for the cell viability assessment, with 500,000 cells being added to each well and the antibiotics added through serial dilution as described (Fig. S1) (Wiegand et al. 2008). Each well in the plate containing antibiotics or antimycotics received 10 μL of CdCl_2_ at a stock concentration of 10,000 μM, resulting in a final concentration of 100 μM CdCl_2_. A CdCl_2_ concentration of 100 μM was selected because it is comparable to the concentration of CdCl_2_ found in Canadian AMD (Olaveson and Nalewajko 2000), and *E. mutabilis* has previously demonstrated tolerance to this concentration of CdCl_2_ under the conditions used in this experiment (Kennedy et al. 2023). One well in each plate was a CdCl_2_ control containing only CdCl_2_ at a concentration of 100 μM and cells. Culture plates were sealed with parafilm and incubated in the environmental chamber under the same conditions (16:8 LD cycle at 260 µmol s^−1^ m^−1^; 24℃ ± 0.5℃ in light and 18℃ ± 0.5℃ in dark; shaking at 120 RMP) as the initial antibiotic trials for 72 h. After 72 h assessments of cell count, cell viability, chlorophyll content, and agar plate growth checks were carried out using the methods described.

This method of assessing Cd tolerance, including subsequent analyses, was carried out on *E. mutabilis* and *E. gracilis*. All experiments were performed in triplicate.

### DNA isolation

Total genomic DNA (gDNA) was isolated from cultures of CPCC 657 grown in different media to selectively enhance or limit the growth of *E. mutabilis* and its unidentified bacterial and fungal partners. CPCC 657 was grown in MAM (pH 4.3), MAM with CdCl_2_ at a concentration of 100 μM (pH 4.3), MAM (pH 2.7), MAM with CdCl_2_ at a concentration of 100 μM (pH 2.7), potato dextrose broth (PDB) grown in light, PDB grown in dark, tryptic soy broth (TSB) grown in light, and TSB grown in dark. Cultures were grown for 7 days in the Conviron PGC20 environmental growth chamber under standard aeration (100RPM on a Thermo Fisher Scientific MaxQ 3000) while cycling light and temperature (16:8 LD cycle at 260 µmol s^−1^ m^−1^; 24℃ ± 0.5℃ in light and 18℃ ± 0.5℃ in dark).

After 7 days, 1 mL of culture was aliquoted into a microfuge tube, centrifuged (6000 RCF for 5 min), and media decanted. Total cellular DNA isolations were carried out according to Hoffman and Winston (1987), with modifications. Cells were resuspended in 300 μL of Triton solution and transferred to a 1.5-mL screw-cap tube containing 100 μL of glass beads and 300 μL 25phenol:24chloroform:1 isoamylalcohol. Samples were vortexed for 6 min, then 200 μL of TE was added, and the tubes were centrifuged at 4℃ (maximum speed for 5 min). The aqueous layer was transferred to a fresh 1.5-mL microfuge tube. Samples were precipitated in 1 mL of ice-cold 100% EtOH and centrifuged at 4℃ (maximum speed for 2 min) before decanting the EtOH; 400 μL of TE and 1.5 μL of RNAaseA solution (20 mg/mL) were added to each tube, and samples were incubated at 37℃ for 30 min. After incubation, 10 μL of 4 M ammonium acetate and 1 mL of ice-cold 100% EtOH were added to each tube; they were then mixed, centrifuged at 4℃ (maximum speed for 2 min), and decanted. The pellet was washed with ice-cold 70% EtOH. Following centrifugation, the EtOH was decanted, and the pellets were air-dried and dissolved in 50 μL TE. Samples were visualized on a 0.8% (w/v) agarose (BioShop Canada Inc.) gel in 0.5 × TE with ethidium bromide gel to assess the integrity of the gDNA and quantified using spectrophotometry (Thermo Fisher Scientific Nanodrop 8000 Spectrophotometer).

### 16S and ITS full-length amplicon PacBio sequencing

High-quality gDNA samples (at least 100 ng/μL and a 260/280 ratio of approximately 1.80 as determined using a Thermo Fisher Scientific Nanodrop 8000 Spectrophotometer) were sent to the Integrated Microbiome Resource (IMR, Dalhousie University, Canada) for library preparation and sequencing on a PacBio Sequel II sequencer. Full 16S (forward primer: 27F(Paliy) = 5′-AGRGTTYGATYMTGGCTCAG-3′; reverse primer: 1492R = 5′-RGYTACCTTGTTACGACTT-3′) and full ITS (forward primer: 5′-ITS1FKYO2-3′ = 5′-TAGAGGAAGTAAAAGTCGTAA-3′; reverse primer: ITS4KYO1 = 5′-TCCTCCGCTTWTTGWTWTGC-3′) sequences were obtained and reported in the form of hifi.fastq.gz file types.

### Bioinformatic analysis

16S and ITS sequencing data were processed using the PacBio CCS pipeline (Comeau et al. 2017) of the Quantitative Insights Into Microbial Ecology 2 (QIIME2) v 2022.11 software (Bolyen et al. 2019). The *dada2* algorithm was used to denoise raw sequences (Callahan et al. 2016). Taxonomic classification was performed against the SILVA 138.1 SSU Ref NR99 full-length database for 16S data (Quast et al. 2012) and the ITS custom classifier for all eukaryotes generated from the UNITE database (Comeau et al. 2017), using the SK-Learn command from the q2-feature-classifer plugin (Bokulich et al. 2018). Reads were then rarified to filter out low-depth samples and amplicon sequence variants (ASVs) with frequencies of less than 100, as well as mitochondrial, chloroplast, and unclassified sequences. Resultant tables from QIIME2 were imported into R-Studio for visualization and graphical analysis. The relative abundance of each ASV was plotted using the *phyloseq* package in R (McMurdie and Holmes 2013).

### PCR amplification

PCR amplification was performed in a total reaction volume of 50 μL containing 4 μL of template DNA, 27.5 μL of sterile deionized water, 4 μL of dNTPs, 10 μL of Phusion Buffer HF (Thermo Scientific™), 0.5 μL of Phusion DNA Polymerase (Thermo Scientific™), and 2 μL of each primer (5 μM). The ITS region was amplified (Raja et al. 2017) (forward primer: ITSF = 5′-CTTGGTCATTTAGAGGAAGTAA-3′; reverse primer: ITS4 = 5′-TCCTCCGCTTATTGATATGC-3′), and the 16S region was amplified (Thijs et al. 2017) (forward primer: 68F = 5′-TNANACATGCAAGTCGRRCG-3′; reverse primer: 518R = 5′-WITACCGCGGCTGCTGG-3′). Amplifications were performed with a Veriti™ Thermocycler (Applied Biosystem™), using the following program: denaturation for 30 s at 98 °C; then 35 cycles consisting of 98 °C for 10 s, 61 °C for 30 s, and 72 °C for 30 s; and a final extension step at 72 °C for 10 min. Each sample was amplified by 6 separate reactions with a volume of 50 μL and subsequently pooled for a total sample volume of 300 μL.

### Gel extraction and DNA purification

Each pooled sample was added 60 μL of loading dye, and amplification products were analyzed by electrophoresis in a 0.8% (w/v) agarose (BioShop Canada Inc.) gel in 0.5 × TE with ethidium bromide in order to visualize DNA bands. ITS amplification resulted in a DNA fragment of approximately 460 bp, while 16S amplification resulted in a DNA fragment of approximately 400 bp. The DNA band visualized on the gel at the appropriate location was subsequently cut out and purified using the PureLink™ Quick Gel Extraction Kit (Invitrogen™).

### Sequencing Sanger

Gel-purified DNA was prepared for Sanger sequencing using the BigDye™ Terminator v3.1 Cycle Sequencing Kit with modifications. Template DNA was normalized to approximately 70 ng/μL, and 3.00 μL was added to a 96-well plate (Progene Cat. No. 87-C96-ABI-2) along with 3.20 μL of primer (0.5 μM), 1.33 μL of Ready Reaction (Applied Biosystems™), 1.33 μL of BigDye Buffer (Applied Biosystems™), and 1.14 μL of deionized water for a total volume of 10.00 μL in each well. Six primers were selected for ITS identification (Raja et al. 2017), and 2 primers were used for 16S identification (Thijs et al. 2017) (Table S2). Amplifications were performed with a Veriti™ Thermocycler (Applied Biosystem™), using the following program: denaturation for 1 min at 96 °C; then 40 cycles consisting of 96 °C for 10 s, 50 °C for 5 s, and 60 °C for 4 min. Following amplification, 2.5 µL of 125 mM EDTA and 30 μL of 100% cold EtOH were added to each well. The plate was sealed, inverted 4 times to mix, covered with aluminum foil, incubated at room temperature for 15 min, and then centrifuged for 4℃ (2500 × *g* for 30 min). The plate was then decanted by being placed upside down in a centrifuge and spun (190 × *g* for 60 s); 30 µL of ice-cold 70% EtOH was added to each well. The plate was sealed, inverted 4 times to mix, and then centrifuged for 4℃ (1650 × *g* for 15 min). The plate was then decanted by being placed upside down in a centrifuge and spun (190 × *g* for 60 s); 15 μL of Hi-Di™ Formamide (Applied Biosystems™) was added to each well; the plate was sealed and quickly centrifuged and covered in aluminum foil for a 15-min incubation at room temperature. The plate was run on an ABI 3730 (Applied Biosystems™), and bases were called using SequencingAnalysis v5.4 to produce.phd1 files. The quality of the files was assessed using Seq Scanner 2 (v. 2.0; Applied Biosystems), and the data were assembled into contiguous (contig) sequences using SeqMan Pro (v. 11.2.1; DNASTAR). The average read length ± the standard deviation was assessed for each contig assembly. Generated contigs were then analyzed using Nucleotide BLAST.

## Results

### *Antibiotic treatments and CdCl*_*2*_

After 72 h of growth, control *E. mutabilis* cells arrived at a concentration of approximately 9.4 × 10^5^ ± 6.4 × 10^4^ cells/mL, while cells exposed to 100 μM CdCl_2_ were approximately 5.5 × 10^5^ ± 1.7 × 10^4^ cells/mL (a 41% reduction in cells). Similarly, control *E. gracilis* cells grew to a concentration of approximately 8.0 × 10^5^ ± 8.1 × 10^4^ cells/mL, while cells exposed to 100 μM CdCl_2_ grew to approximately 5.4 × 10^5^ ± 3.3 × 10^4^ cells/mL (a 31% reduction in cells). A *t*-test revealed that the application of 100 μM CdCl_2_ statistically (*p* < 0.05) decreases the number of viable *E. mutabilis* and *E. gracilis* cells in culture, confirming that the application of CdCl_2_ inhibits the growth of *Euglena*.

Cell counts of *E. mutabilis* and *E. gracilis* following antibiotic exposure revealed influences of antibiotics on algal growth (Table S1). Applications of kanamycin, rifampicin, chloramphenicol, and amphotericin B produced similar growth responses in both *Euglena* species. Neither *Euglena* sp. was affected by kanamycin, as evidenced by the lack of statistical (*t*-test; *p* < 0.05) difference in cell counts, while rifampicin, chloramphenicol, and amphotericin B resulted in significant decreases in cell counts at concentrations of 8–64 µg/mL. Notably, the influence of the penicillin–streptomycin blend and tetracycline differed between *E. mutabilis* and *E. gracilis*. Treatment with the penicillin–streptomycin blend here showed a significant reduction in *E. gracilis* cell counts across all concentrations; however, these antibiotics did not have a significant impact on *E. mutabilis* cell counts. This was also seen with the application of tetracycline to *E. gracilis*, which displayed statistical decreases in cell counts across all concentrations of tetracycline, while *E. mutabilis* was only significantly impacted at 8 and 64 µg/mL concentrations.

**Fig. 1:**
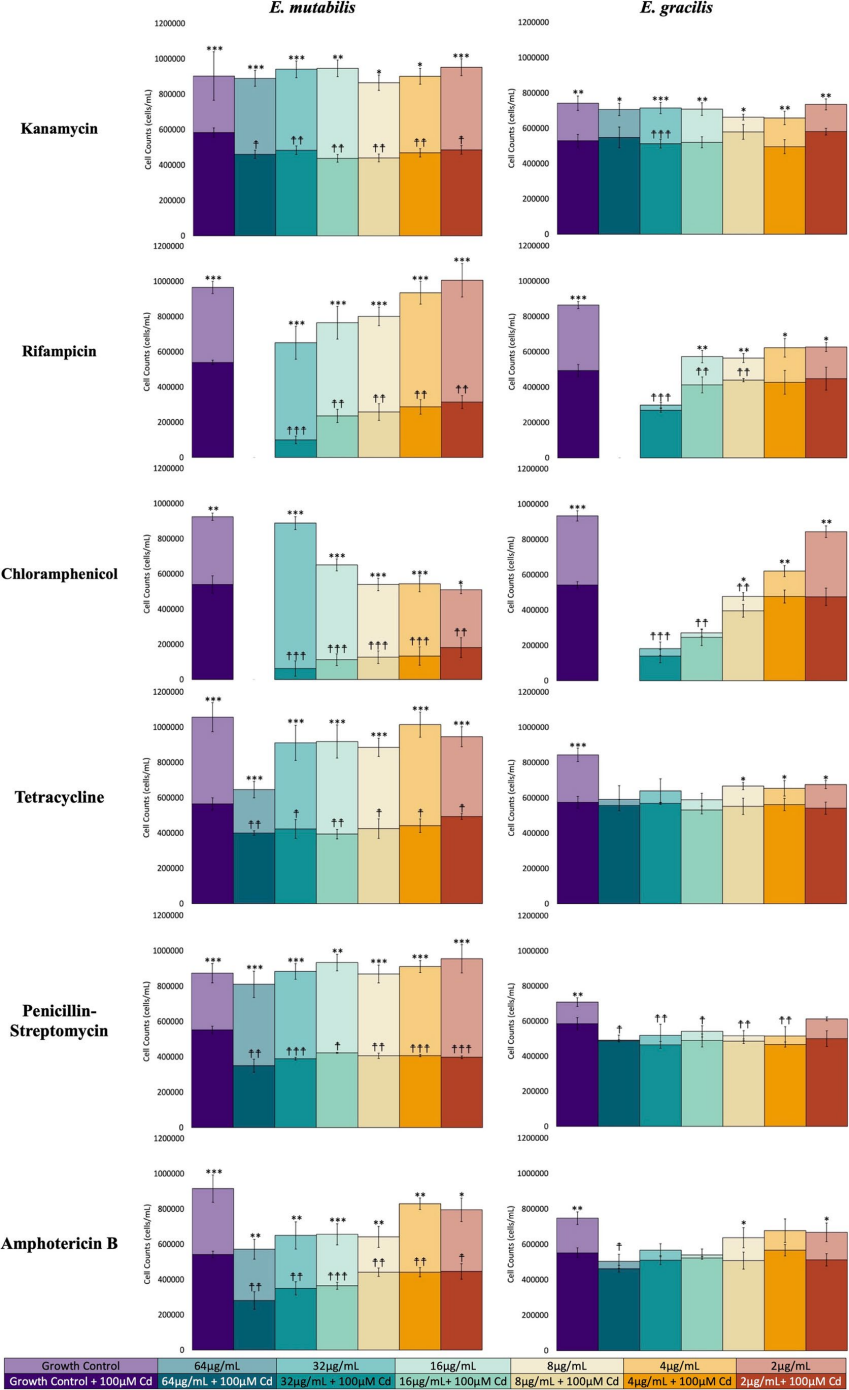
Antibiotic and CdCl_2_ treatment reveal differences in cell viability between *E. mutabilis* and *E. gracilis*. Cultures were grown in MAM for 72 h. Light-colored bars indicate the inclusion of a dilution series of antibiotics alone, while darker-colored bars indicate exposure to antibiotics at a fixed concentration, 100 μM, of CdCl_2_. Colors indicating different antibiotic concentrations are indicated in the legend at the bottom of the figure. Error bars represent standard deviation (*n* = 3). A star (*) indicates statistical difference (*t*-test) between cells exposed to antibiotics and cells exposed to the same concentration of antibiotics with the addition of 100 μM CdCl_2_ (*p < 0.05; **p < 0.01; ***p < 0.001). A dagger (^☨^) indicates statistical difference (*t*-test) between control cells exposed to 100 μM CdCl_2_ and those exposed to antibiotics and CdCl_2_ (^☨^*p* < 0.05, ^☨☨^*p* < 0.01, ^☨☨☨^*p* < 0.001)

Although some antibiotics impacted the growth of *E. mutabilis* and *E. gracilis*, the combination of antibiotics and CdCl_2_ reveals more substantial differences in response between the organisms (Fig. 1). The number of viable cells in *E. mutabilis* treated with both 100 μM CdCl_2_ and any concentration of antibiotics was significantly less than cells treated with only antibiotics (*t*-test*, p* < 0.05; denoted by * in Fig. 1). This was seen across all treatments of antibiotics and antimycotics in *E. mutabilis*. In contrast, statistical decreases in *E. gracilis* cells under the same treatments were only observed with a select concentration of antibiotic treatment (Fig. 1). The addition of 100 μM CdCl_2_ resulted in decreases in viable *E. mutabilis* cells ranging from 31 to 92%, while decreases in *E. gracilis* cells ranged from 1 to 44%. The biggest differences in cell counts were observed in samples treated with chloramphenicol and 100 μM CdCl_2_, which resulted in decreases in cell counts ranging from 64 to 92% in *E. mutabilis* and 9 to 44% in *E. gracilis*, compared to cells treated with only chloramphenicol. Additionally, *E. mutabilis* cells treated with antibiotics and 100 μM CdCl_2_ resulted in statistical decreases in cell counts compared to *E. mutabilis* cells treated with only 100 μM CdCl_2_ (*t*-test*, p* < 0.05; denoted by a dagger (^☨^) in Fig. 1). This result was observed for every concentration of antibiotic and antimycotic application in *E. mutabilis*. This differed from *E. gracilis*, where approximately one-third of the antibiotic treatments revealed a decrease. A final note of the differences between species is that, while there was no difference between the number of viable cells in *E. mutabilis* and *E. gracilis* when exposed to 100 μM CdCl_2_, the number of *E. mutabilis* cells was higher across every concentration of antibiotic in combination with 100 μM CdCl_2_. This suggested that the addition of antibiotics had a greater impact on the CdCl_2_ tolerance of the *E. mutabilis* co-cultures compared to those of axenic *E. gracilis*.

The antibiotics that resulted in the greatest decrease in *Euglena* cell viability were rifampicin and chloramphenicol, for which 64 μg/mL treatments resulted in no viable *E. mutabilis* or *E. gracilis* cells (Fig. 1). However, the pattern of response to different concentrations of chloramphenicol was distinct for the two species. Treatment with 32 μg/mL of chloramphenicol revealed the greatest number of viable *E. mutabilis* cells, which remained as high as control conditions, while the least number of viable cells was seen at a concentration of 2 μg/mL. By contrast, the lowest number of viable *E. gracilis* cells was observed at 32 μg/mL, while the greatest number appeared at 2 μg/mL. When *E. mutabilis* was treated with 100 μM CdCl_2_, it displayed the same trend as *E. gracilis*.

### *Evaluation of chlorophyll content after antibiotic treatments and CdCl*_*2*_

**Fig. 2:**
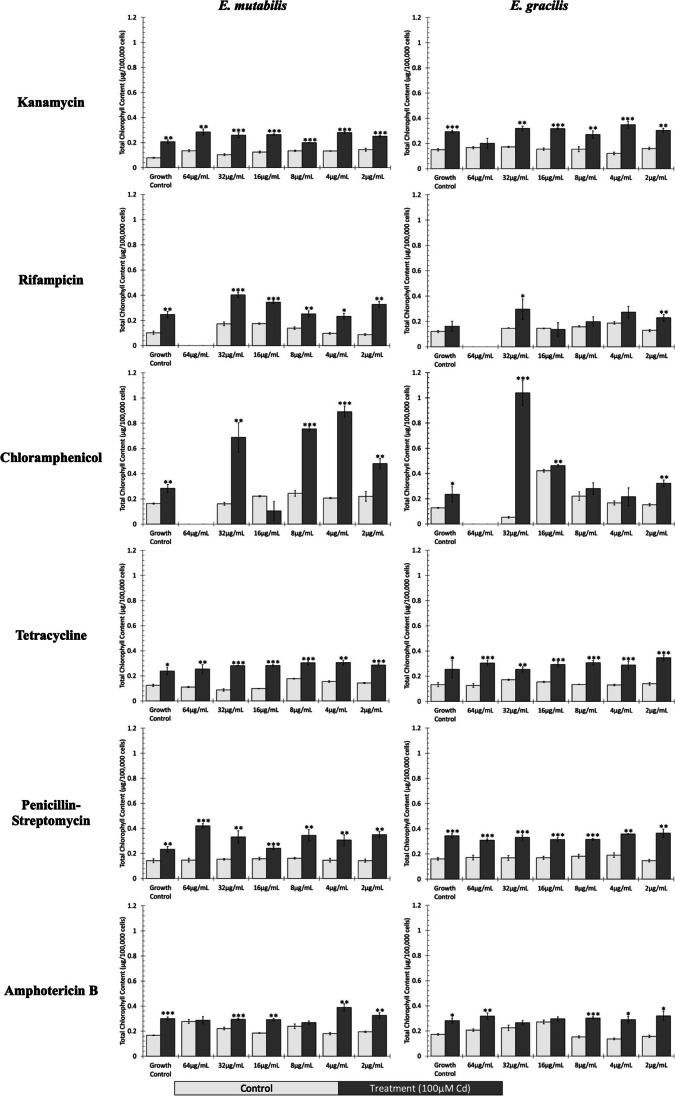
Cadmium exposure increases the chlorophyll content of *Euglena* cultures. Total chlorophyll content (chl *a* + chl *b*) per 100,000 *Euglena* cells was determined after 72 h of exposure to varying concentrations of antibiotics (light-colored bars) and varying concentrations of antibiotics + 100 μM CdCl_2_ (dark-colored bars). Error bars represent standard deviation (*n* = 3). The statistical difference between total chlorophyll content in control and treatment conditions was assessed using a *t*-test, and significant differences are denoted by a star (*p < 0.05; **p < 0.01; ***p < 0.001)

The total chlorophyll content (chl *a* + chl *b*) of *E. mutabilis* and *E. gracilis* cultures treated with antibiotics and 100 μM CdCl_2_ was on average 1.95- and 1.82-fold higher, respectively, than cells only treated with antibiotic (Fig. 2). When exposed to kanamycin, rifampicin, tetracycline, or penicillin–streptomycin, the chlorophyll content of both *E. mutabilis* and *E. gracilis* is below 0.2 μg/100,000 cells; however, upon the addition of 100 μM CdCl_2_, the concentration of chlorophyll significantly increases across every antibiotic concentration. An exception to the impact of antibiotic treatments was the exposure of *E. mutabilis* to amphotericin B, which resulted in an increased chlorophyll content; however, when treated with CdCl_2_ and amphotericin B, the level of chlorophyll was significantly higher than the treatment with antimycotic alone. In contrast to these similar responses, *E. mutabilis* showed increased chlorophyll concentration at all concentrations of rifampin, while the *E. gracilis* response was variable. Furthermore, both species showed variability in the level of chlorophyll present across the various concentrations of chloramphenicol with CdCl_2_, but chlorophyll production was much higher in *E. mutabilis*.

### *Growth recovery following antibiotic treatments and CdCl*_*2*_* exposure*

Recovery of *Euglena* and the fungus co-cultured with *E. mutabilis*, following growth in antibiotics or antimycotics in the presence or absence of CdCl_2_, was assessed by plating on non-selective media. The plates were incubated in the dark for 7 days, leading to only heterotrophic growth. The growth of bacterial colonies was too numerous to count, often exhibiting confluent growth, and therefore colony numbers were not recorded. Intimate growth was observed between colonies of *E. mutabilis* and the fungus (Fig. S2). The control for the experiment was phototrophic growth in MAM ± 100 µM CdCl_2_ without antibiotics, followed by heterotrophic growth on either R2A or PDA plates. In the control, there were large numbers of *E. mutabilis* colonies, but no fungal CFUs (Table 1). Generally, *E. mutabilis* cells treated with antibiotics exhibited decreased recovery compared to control growth, except for tetracycline. Phototrophic growth in MAM + CdCl_2_, followed by heterotrophic growth, exhibited both *E. mutabilis* and fungal colonies. Pre-growth in the presence of antibiotics, with and without CdCl_2_, led to reduced numbers of colonies for both *Euglena* species for most of the antibiotics. An exception to this was *E. mutabilis* pre-grown with tetracycline alone, which did not affect heterotrophic growth, while pre-growth with tetracycline and CdCl_2_ did. A notable pattern in fungal recovery growth response was the reduced recovery following growth in kanamycin and amphotericin B indicating both treatments had a negative impact on fungal viability, but this impact did not occur when CdCl_2_ was present.

**Table 1:** Cell viability test (*n* = *3*) comparing colony-forming units of *E. mutabilis* (CPCC 657) and uncharacterized fungal growth, in addition to *E. gracilis* (CPCC 95) after 7 days of incubation at 24 °C in darkness

	*E. mutabilis*		*Fungi*		*E. gracilis*	
	Media only	100 µM CdCl_2_	Media only	100 µM CdCl_2_	Media only	100 µM CdCl_2_
Control	+ + +	+ +	−	+ + +	+ +	+ + +
	32 μg/mL	32 µg/mL + 100 µM CdCl_2_	32 μg/mL	32 μg/mL + 100 µM CdCl_2_	32 μg/mL	32 µg/mL + 100 µM CdCl_2_
Kanamycin	+ +	+	+	+ + +	+ +	+ + +
Rifampicin	+	+	+ + +	+ + +	+	+
Chloramphenicol	+	+	+ + +	+ + +	+	+ +
Tetracycline	+ + +	+	+ + +	+ + +	+	+ +
Penicillin–streptomycin	+ +	+	+ + +	+ + +	+	+ +
Amphotericin B	+ +	+	+	+ + +	+	+ + +

Bacterial CFUs were excluded as they were too numerous to count.

“ − ” no CFU; “ + ” if < 50 CFU; “ +  + ” if 50 >  < 150 CFU; “ +  +  + ” if > 150 or complete lawn present and CFU count impossible.

### PacBio sequencing and culture identification

**Fig. 3:**
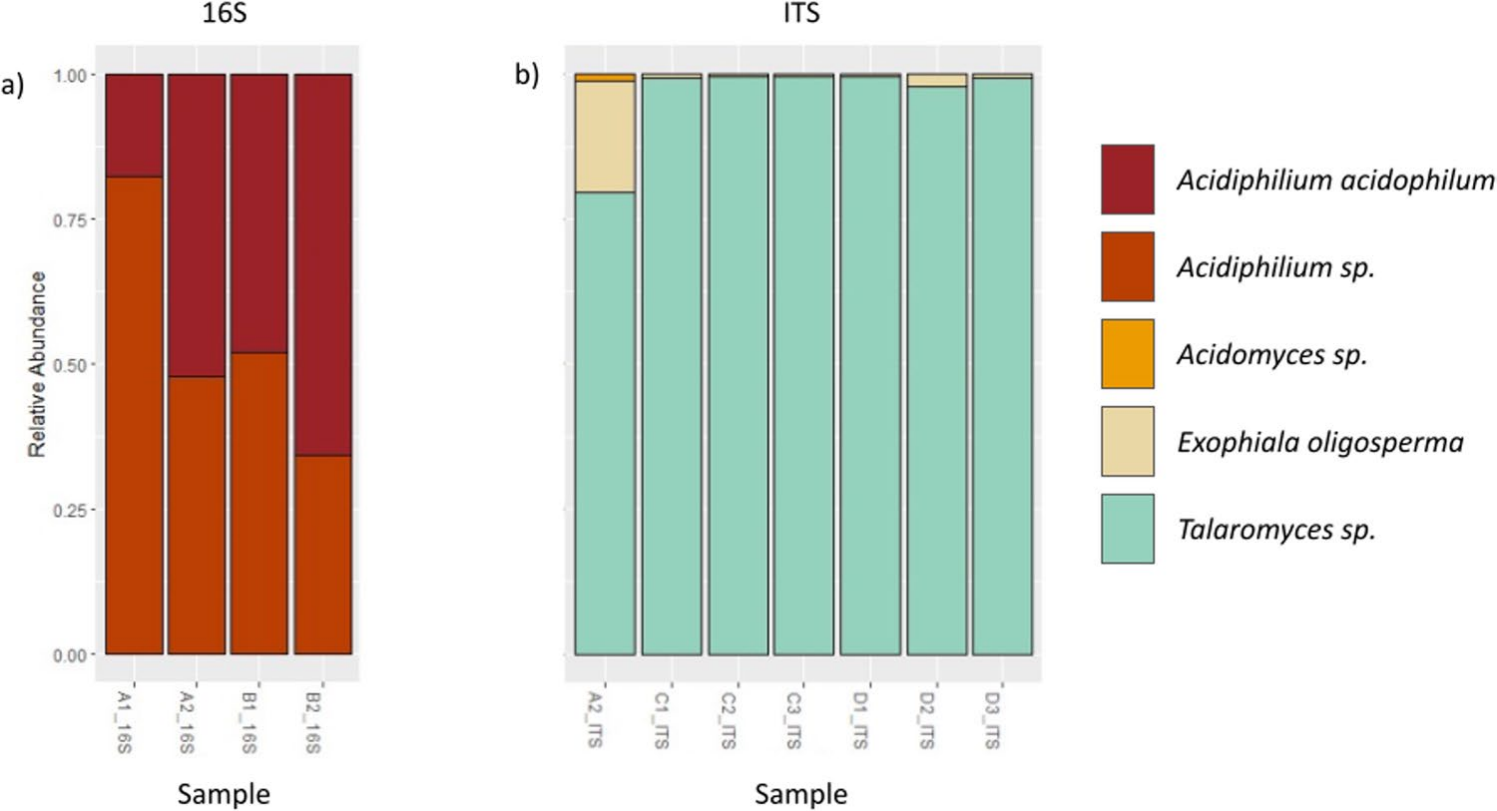
PacBio sequencing identifies a major bacterial and a major fungal partner for *E. mutabilis*. Relative abundance taxa bar plot of *E. mutabilis* co-culture (CPCC 657) grown in MAM at pH 4.3 (A samples), MAM at pH 2.7 (B samples), TSB grown in light (C samples), and TSB grown in dark (D samples). Taxa (targeting the full 16S region of bacterial rDNA and full ITS region of fungal rDNA) are represented at the species level where possible

A total of 372,830 reads were obtained from sequencing the full-length 16S ribosomal RNA (rRNA) gene. Reads per sample ranged from 7297 to 62,135. Sequencing the full-length internal transcribed spacer (ITS) gene obtained a total of 154,027 reads, ranging from 165 to 29,136 per sample. Denoising left 229,321 reads (62%), with an average of 22,932 sequences per sample for 16S, and 141,901 reads (92%), with an average of 14,190 sequences per sample for ITS. Filtering of low-level amplicon sequence variants (ASVs), low-depth samples, as well as unclassified, chloroplast, and mitochondrial sequences resulted in the exclusion of six samples and reserved a total of 34,857 reads (9%), with an average of 8714 reads per sample for 16S. The filtering process resulted in the exclusion of three samples from ITS and reserved 140,242 sequences (91%), averaging 20,034 sequences per sample. The sequences that passed the denoising and filtering processes were clustered into four ASVs for 16S and three ASVs for ITS. In total, 91% of the 16S sequences were excluded due to low sample depth or low frequency or were classified as mitochondrial, chloroplast, or unclassified at the genus level. Of these, 187,716 sequences were classified as *Euglena*, alluding to the overrepresentation of this contributor*.* 16S sequencing identified one bacterial genus, *Acidiphilium* (Fig. 3a). Of the sequences classified at the genus level as *Acidiphilium*, 46% were further classified at the species level as *Acidiphilium acidophilum*. The remaining sequences could not be classified at the species level. Thus, the major bacterial contributor is determined to be *Acidiphilium acidophilum*. ITS sequencing identified three fungal genera: *Acidomyces*, *Exophiala*, and *Talaromyces* (Fig. 3b); only one ASV could be classified to the species level (*Exophiala oligosperma*). Of the total ITS sequences that passed filtering, 98% were classified as *Talaromyces*, whereas *Exophiala* and *Acidomyces* contributed only 2.13% and 0.09% of sequences, respectively. Thus, the major fungal contributor is determined to be a species of the genus *Talaromyces.*

### Sanger sequencing results

Sanger sequencing for ITS samples generates high-quality reads of 235 ± 119 bp, with initially ambiguous bases being manually called. *Talaromyces amestolkiae* showed a 93.63% match with the raw data (562 bp length), which was increased to 94.64% upon manually calling ambiguous bases (565 bp length). The 16S reads generated were of medium quality, with a mean ± standard deviation of 151 ± 27 base pairs. The contig assembly produced one single contig with a length of 281 bp, approximately 62% of the target region. The top match for the sequence was a 94.58% match to an uncultured bacteria isolated from acidic soils, while the next result was a 94.22% match to *Acidiphilium* sp.

## Discussion

The Cd tolerance of an *E. mutabilis* natural co-culture with unknown fungi and bacteria was investigated. Although extensive attempts were made, the culture bank was unable to create an axenic *E. mutabilis* culture from any natural isolate. Therefore, we used an axenic *E. gracilis* culture as a control for assessing the impact of antibiotic treatments on the *E. mutabilis* co-culture. *E. gracilis* is the model euglenoid and has been the focus of antibiotic and HM experiments using a euglenoid (Bishop and Smillie 1970; Gumińska et al. 2018; He et al. 2021; Shao et al. 2018). A recent investigation revealed *E. mutabilis* had greater tolerance to CdCl_2_ than *E. gracilis*, and that it responded differently to pre-growth under nutritional conditions (Kennedy et al. 2023). The cultures were grown in the presence of antibiotics with and without CdCl_2_. Comparing the results between the two species allowed us to gain insight into the other microbes growing with *E. mutabilis*.

Cultures were treated with five antibiotics and two antimycotics. There was no impact on the viability of *E. gracilis* or *E. mutabilis* when grown in the presence of kanamycin; however, the viability of *E. mutabilis* decreased in the presence of CdCl_2_ and kanamycin, while *E. gracilis* viability was not influenced by this treatment. These results were consistent with previous research investigating *Euglena* sp. responses to kanamycin (He et al. 2021; Shao et al 2018) and indicated that any impact of kanamycin on the chloroplast or mitochondria of these *Euglena* species did not influence their viability. Therefore, the impact of kanamycin on CdCl_2_ tolerance can be attributed to kanamycin inhibiting the translation of a culture member that is providing CdCl_2_ tolerance to the consortium. Furthermore, since kanamycin is active against gram-negative bacteria with little impact on gram-positive bacteria (Zeng et al. 2019) and no impact on fungi (Umezawa et al. 1957), this suggests that the consortium enhancing tolerance is a gram-negative bacterium.

The viability of both *Euglena* species was zero at 64 μg/mL rifampin, while other rifampin concentrations had a greater impact on *E. gracilis* viability than that of *E. mutabilis*. We propose that this impact on viability was likely due to a negative impact on organelle transcription (McClure and Cech 1978). In the presence of CdCl_2_, both *Euglena* species have reduced viability at a rifampin concentration of up to 8 μg/mL, which may suggest that organelle transcription provides a component of the protection against the CdCl_2_ challenge. This is consistent with earlier research indicating chloroplasts in *E. gracilis* play a role in CdCl_2_ tolerance (Khatiwada et al. 2020a).

Chloramphenicol at 64 μg/mL kills all *Euglena* cells, likely through its inhibition of organelle translation (Choi et al. 2020). Chloramphenicol has also been shown to inhibit the synthesis of ribulose diphosphate carboxylase (RuBisCO) and NADP-linked glyceraldehyde-3-phosphate dehydrogenase (NADP-GAPDH) in *Euglena* (Hovenkamp-Obbema and Stegwee 1974). These carbon fixation enzymes are located in the chloroplast, and their inhibition is a potential aspect of reduced chloroplast function caused by chloramphenicol (Davis and Merrett 1975; Hovenkamp-Obbema and Stegwee 1974), which could negatively influence Cd tolerance. Interestingly, the viability of *E. mutabilis* decreases with decreasing chloramphenicol concentrations, while the viability of *E. gracilis* increases with decreased chloramphenicol concentrations. This unexpected difference in response may be due to the presence of other consortium organisms in the *E. mutabilis* culture. The presence of chloramphenicol-metabolizing enzymes in some bacteria enables them to inactivate and breakdown the antibiotic, using it as a carbon source (Kim et al. 2019a; Nakano et al. 1977). An *E. mutabilis* FAB consortia organism with this function could influence the response of the culture to chloramphenicol contributing to the observed dose curve response.

Previous studies have shown that *E. gracilis* can tolerate tetracycline concentrations ranging from 100 to 300 μg/mL (Ebringer et al. 1996; Shao et al. 2018). The results here indicate that tetracycline has an impact on *E. gracilis* viability at a concentration as low as 2 μg/mL, which does not align with previous research, whereas the impact on *E. mutabilis* viability at all concentrations was not significant except at 8 μg/mL. However, tetracycline significantly reduces *E. mutabilis* viability in the presence of CdCl_2_ while its influence on *E. gracilis* viability in the presence of CdCl_2_ is not significant. This suggests that tetracycline is inhibiting the translation of a consortium member in the *E. mutabilis* culture (Nguyen et al. 2014). The broad-spectrum activity of tetracycline does not allow the nature of this consortium member to be determined.

Penicillin and streptomycin are antibiotics that have repeatedly been shown to bleach *E. gracilis* (Ebringer et al. 1969; Ebringer et al. 1970; He et al. 2021; Zahalsky et al. 1962). The results here indicate that the addition of a combination of penicillin and streptomycin does not affect the viability of *E. mutabilis*, but does have an influence on *E. gracilis* viability; however, the combined antibiotics had a significant impact on the viability of *E. mutabilis* exposed to CdCl_2_. There was no impact of the combined antibiotics on *E. gracilis* CdCl_2_ tolerance. This suggested that these antibiotics are inhibiting a consortium member that influences the CdCl_2_ tolerance of the *E. mutabilis* culture. While penicillin has a greater effect on gram-positive bacteria, streptomycin is a broad-spectrum bacteriostatic that may also impact fungal growth (Schneck et al. 1971), so the nature of the consortium member influenced by this treatment cannot be determined.

The viability of both *Euglena* species was affected by amphotericin B; however, the impact of amphotericin B treatment on Cd sensitivity was consistently significant only for the *E. mutabilis* culture. The greater impact on Cd tolerance in *E. mutabilis* is supported by the results, in that a consortium member—specifically, a fungus—likely aids in the Cd tolerance of the *E. mutabilis* culture. While all these results could reflect differences in *E. mutabilis* responses relative to *E. gracilis*, we note that one major difference between the cultures is the presence of other microbes. As such, we propose that these analyses support a role for the microorganisms growing with *E. mutabilis* in the tolerance of the culture to CdCl_2_ and suggest that the organisms supporting CdCl_2_ tolerance are a gram-negative bacterium and a fungus susceptible to amphotericin B.

The presence of microbes assisting a photobiont (algae, cyanobacteria, or Euglenoid) is numerous. Many algae rely on exogenous vitamin B_12_ (cobalamin) and nitrogen, which can be supplied by co-cultured bacteria (Croft et al. 2005; Kazamia et al. 2012; Liba et al. 2006) in exchange for the bacteria receiving a carbon source from the algae (Amin et al. 2015; Seymour et al. 2017). Furthermore, the growth of the marine diatom *Ditylum brightwellii* in nitrogen-limiting media and its symbiotic bacterium in carbon-limited media are both lower relative to co-cultures (Brussaard and Riegman 1998). Similarly, an investigation of a symbiotic culture of *Chlorella vulgaris* and *Bacillus subtilis* revealed that co-culturing resulted in increased growth, photosynthetic activity, carbon fixation, and vitamin B_12_ content of the alga (Kranner et al. 2005).

Examples of the influence of fungi include the finding that fungi cultured with algae provide nutrients as well as mechanisms for protection for the algae (Grube and Wedin 2016; Kranner et al. 2005; Krespach et al. 2020). When *Chlamydomonas reindhardtii* was cultured with *Aspergillus nidulans* and exposed to the algicide azalomycin F, algal cells grew within fungal hyphae, avoiding contact with the algicide (Krespach et al. 2020). Additionally, the presence of azalomycin F prompted *A. nidulans* to produce polar lipids, which attract the algicide and effectively neutralize it (Krespach et al. 2020). Fungi also provide protection from reactive oxygen species (ROS) generated during HM exposure and produce extracellular polymeric substances (EPS) and organic acids that can bind HMs, reducing their toxicity when co-cultured with algae (Park et al. 2005; Shen et al. 2018; Wang et al. 2022). Further examples that are more directly related to the potential biotechnological application of the results here come from research that shows several algae have been investigated for bioremediation of textiles, organic pollutants, and HMs (Rehman 2011; Salgueiro et al. 2016; Waluyo et al. 2020; Znad et al. 2018), and recent studies have suggested that co-culturing algae with bacteria or fungi provides benefits to these applications. These include increased flocculation efficiency, increased biomass, independent nutrient exchange, and enhanced tolerance to extreme environments (Ayangbenro et al. 2019; Fuentes et al. 2016; Lal et al. 2021; Mujtaba et al. 2015; Nazari et al. 2020; Zhang et al. 2021). The creation of artificial associations with algae, fungi, and bacteria has also been used to enhance heavy metal uptake (Wang et al. 2021), and the model euglenoid *E. gracilis* has been co-cultured with other microbes to enhance nutrient availability (Lukáčová et al. 2022; Rubiyatno et al. 2021). Given these previously described examples of microbes assisting photobionts, it is not unreasonable to propose that the data presented here supports a role for bacterial and fungal species as members of a FAB consortium with *E. mutabilis*, and that they contribute to the Cd tolerance of this consortium.

Although we observed a decrease in the number of viable *Euglena* cells following treatment with antibiotics and CdCl_2_, there was an increase in chlorophyll production by *Euglena* in the presence of Cd (Fig. 2). This was unexpected because Cd is known to disrupt the physiological and metabolic processes of phototrophic organisms, including algae, cyanobacteria, and plants, primarily by reducing the photosynthetic rate and chlorophyll concentration (Andresen and Küpper 2013; Choppala et al. 2014; Du et al. 2019a; Jamers et al. 2013; Trevors et al. 1986). Both *E. mutabilis* and *E. gracilis* display significantly greater chlorophyll content per 100,000 cells under Cd exposure. Furthermore, the amount of chlorophyll being produced in the presence of Cd appears unaffected by the addition of antibiotics, suggesting that the impact does not affect the associated bacteria. This phenomenon has been reported in higher plants that have taken up Cd from their surroundings (Grajek et al. 2020; Zhou and Qiu 2005). The structural foundation of a chlorophyll molecule is Mg; however, Mg is also essential for several other metabolic processes, including enzyme activation, sucrose transport, and energy metabolism (Ishfaq et al. 2022). It has been shown that Cd can replace Mg in the central position of a chlorophyll molecule as they are both divalent metals (Grajek et al. 2020). Furthermore, the Cd hyperaccumulator *Sedum alfredii* demonstrated no noticeable reduction in photosynthetic activity and a simultaneous increase in chlorophyll content following Cd treatments, despite negative impacts on leaf and root growth (Zhou and Qiu 2005). A similar result was observed in *Chlamydomonas reinhardtii* under excess Cu exposure, which is also a divalent metal (Jiang et al. 2016). A correlation was observed between increased chlorophyll and cell survivability, which was suggested to be the result of chlorophyll accumulation in cells that do not divide (Jiang et al. 2016). Here we showed that Cd treatment of *Euglena* led to a reduction in cell viability and an increase in the amount of chlorophyll produced per cell. This could indicate that although exposure to divalent HMs debilitates overall culture health, Cd may be able to replace the Mg in chlorophyll, leaving a less efficient but more plentiful photosynthetic apparatus that allows the *Euglena* to survive some HM toxicity.

Despite the increasing chlorophyll content per cell, *E. mutabilis* is unable to recover from antibiotic and Cd exposure during subsequent heterotrophic growth (Table 1). The effects of antibiotics on *E. gracilis* have been extensively studied (Ebringer et al. 1969; Ebringer 1972; Ebringer et al. 1996; He et al. 2021; Scott 1976; Zahalsky et al. 1962), and the results here are consistent with previous findings; however, there has been no work on the impact of antibiotics on *E. mutabilis*. Based on CFU comparisons, *E. mutabilis* recovers after antibiotic exposure better than *E. gracilis*, but *E. gracilis* shows better recovery following treatment with antibiotics and Cd. This is notable because a synergistic effect can occur where toxicity is increased through the formation of antibiotic and divalent HM complexes (Fu et al. 2023; Khurana et al. 2021). The increased chlorophyll content in the presence of Cd suggests that both *Euglena* species can generate energy and survive the treatment. Upon transfer to nutrient-rich media plates, both species could shift to heterotrophic growth and employ a number of ways to recover from Cd stress, including the use of major facilitator superfamily (MFS) transporters, transmembrane (TrkA) transporters, and HM pumps (P1 B ATPase) (Grenson 1964; Khatiwada et al. 2020b). We postulate that while both *Euglena* species can recover, the recovery by the fungi and bacteria co-cultured with *E. mutabilis* occurs more quickly than that of *E. mutabilis;* consequently*,* substantial fungal and bacterial growth in heterotrophic conditions results in fewer *E. mutabilis* colonies are being formed.

The fungi and bacteria in the *E. mutabilis* co-culture recovered well following antibiotic and CdCl_2_ exposure and often grew to take over the entire plate. In heterotrophic cultures containing fungi and microalgae, a similar overgrowth by the fungus was previously observed (Bansfield et al. 2022; Bhattacharya et al. 2017; Gultom and Hu 2013; Wyatt et al. 2019). The observations that extensive fungal colonies were visible following heterotrophic growth after antibiotic exposure and not while the culture was previously grown under phototrophic conditions in MAM suggest that antibiotic and CdCl_2_ treatment suppressed bacterial growth and allowed the fungus to access nutrients typically taken up by the bacteria, thus giving it an improved ability to recover during heterotrophic growth. However, the results show that the number of fungal CFUs decreases with the concentration of antibiotics (Table S3), suggesting that the fungal-bacterial interaction is not simply a matter of presence or absence. Together, these results suggest that co-cultures, in which the carbon source comes from *E. mutabilis* photosynthesis requires a specific light regimen and organism ratio to ensure the fungus and/or bacteria do not overwhelm the photobiont. Interactions between the fungus and the bacteria were further informed by determining the species present.

Sequence analysis of DNA extracted from the *E. mutabilis* FAB co-culture was used to identify the prominent constituent organisms present in the co-culture. The fungus was determined to belong to the genus *Talaromyces*, with a possible species identification being *T. amestolkiae*, while the bacteria were determined to belong to the genus *Acidiphilium*, with a probable species identification being *A. acidophilum* (Fig. 3). Consistent with the fungus belonging to *Talaromyces* was the observation of yellow and red fungal colonies on PDA plates following amphotericin B and cycloheximide exposure (Fig. S3) (Morales-Oyervides et al. 2020). Sequence analysis also detected a low-level sequence with similarity to *Exophiala oligosperma*; however, this may have been an artifact since *E. oligosperma* colonies are black in color, and there is no evidence that *E. oligosperma* can tolerate HMs (Kennes and Veiga 2004; Tokuhisa et al. 2011). *Talaromyces* sp., on the other hand, have been characterized by their ability to produce different colored pigments based on the carbon source present and in response to stress or predators (Koolen et al. 2013; Morales-Oyervides et al. 2020; Yilmaz et al. 2012). Furthermore, they have been isolated from HM-polluted areas and can withstand high concentrations of Cr, As, Pb, Ni, Cu, and Cd (Guerra Sierra et al. 2022; Nam et al. 2019; Priyanka and Dwivedi 2023). Red pigment is produced by fungi that are susceptible to cycloheximide and carry the recessive *ade2* gene, which, when repressed, results in red pigmentation from the accumulation of phosphoribosylaminoimidazole, an intermediate in the biosynthesis of adenine (Ajimura et al. 1993; Granot and Snyder 1993; Mano et al. 2013; Parry and Zimmerman 1976; Vyas et al. 2015). The yellow pigmentation following amphotericin B and cycloheximide exposure is most likely composed of products of the azaphilone family, namely mitorubrinol and mitorubrinic acid (Morales-Oyervides et al. 2020; Tam et al. 2015; Woo et al. 2012). These compounds are found in numerous *Talaromyces* sp. and are proposed virulence factors regulated by polyketide synthesis genes *pks11* and *pks12*, which become activated under stress (Morales-Oyervides et al. 2020; Tam et al. 2015; Wang et al. 2023; Woo et al. 2012). *Talaromyces* sp. are known to be susceptible to amphotericin B, which may activate the *pks11* and *pks12* genes and cause the pigmentation seen during heterotrophic growth (Sheng et al. 2021). This cumulative information, in combination with confirmatory Sanger sequencing results, indicates that the predominant fungus present in the *E. mutabilis* co-culture is a *Talaromyces* sp.

The production of pigments by *Talaromyces* sp. only when stressed or acting as a pathogen suggests that fungus in co-culture, where it does not produce pigments, is not stressed or acting as a pathogen. Instead, we propose that the fungus has a positive role in the FAB co-culture. Based on what is known about *Talaromyces* sp., we can attribute several possible impacts provided by the fungal consortium member. The fermented broth of red pigmentation produced by *T. amestolkiae* has shown antimicrobial activity against *Staphylococcus aureus*, although the pigmentation was significantly less cytotoxic to gram-negative bacteria and displayed low cytotoxicity against fibroblasts NIH.3 T3 (Mussagy et al. 2023; Zaccarim et al. 2019). If the consortium came under stress from an invading gram-positive bacterium, *Talaoromyces* pigment production may offer protection. However, since no pigment formation was noted in the co-culture, the *Talaromyces* antibiotic production did not likely affect the experiments carried out here. *Talaromyces* sp. are also known to produce the plant growth-promoting hormone indole-3-acetic acid (IAA), and they display tolerance to Cd when growing in soil (Xiao et al. 2023). As such, when this fungus was cultured with *Arabidopsis thaliana*, it reduced the amount of Cd in the soil and underground plant tissues while simultaneously increasing plant growth. It was postulated that the fungus promoted nutrient uptake and IAA production to promote plant development (Emery et al. 1998) and provided protection by increasing the essential nutrient bioavailability under low Cd concentrations, thereby effectively diluting the presence of Cd and enhancing plant HM tolerance (Xiao et al. 2023). These mechanisms are prevalent during other plant-fungal interactions (Sudová and Vosátka 2007; Velivelli et al. 2014; Yang et al. 2021) and may be fundamental to stress response in algal-fungal symbiosis (Du et al. 2019b; Li et al. 2019; Sudová and Vosátka 2007; Wang et al. 2022), as well as being present in the *Euglena*–*Talaromyces* interaction. Therefore, the *Talaromyces* sp. in the consortium may be providing protection from gram-positive bacteria, enhanced Cd tolerance and it may enhance the *E. mutabilis* growth.

Finding that the predominant bacterial species in the FAB co-culture is an *Acidiphilium* species is consistent with this species being found in acidic environments with high HM concentrations (Li et al. 2020; Xu et al. 2021; Yang et al. 2020). *A. acidophilum* is an obligate heterotroph that could act as a nutrient source for other organisms in the FAB co-culture as it is a facultative, sulfur-reducing mixotroph (Johnson 2009; Rawlings 2002; Priya and Hait 2020); however, there is only evidence of heterotrophic growth in AMD (Johnson and Bridge 2002; Liu et al. 2011). When growing heterotrophically, the main energy source for *A. acidophilum* is ferrous iron, which it can reduce to generate a usable form (Liu et al. 2011; Priya and Hait 2020) and is abundant in AMD (Akcil and Koldas 2004). *A. acidophilum* is a gram-negative bacterium and therefore less susceptible to the potential cytotoxic effects of the *Talaromyces* member of the consortium. In the presence of fungal-produced gram-positive affecting antibiotics, *A. acidophilum* would have a competitive advantage. Cytotoxicity effects of *A. acidophilum* have not been reported. The production of an antibiotic by the fungal component of the consortium and a carbon source by the photobiont *E. mutabilis* suggests benefits for *A. acidophilum* in the association. The results of the antibiotic experiments suggest that, in return, *A. acidophilum* contributes to the Cd tolerance and possibly other stresses the consortium is exposed to.

The characteristics of *Talaromyces* sp*.* and *A. acidophilum* are consistent with their persistence in the FAB co-culture and suggest they have a role in the stress protection of and nutrient exchange with *E. mutabilis*. If the production of compounds by the fungal member of the consortium is confirmed and they are shown to elicit an effect on other members of the consortia, then this association would be interpreted as a commensal or holobiont type of interaction (Berlanga-Clavero et al. 2020; Krespach et al. 2020; Rasmann and Turlings 2016; Wooldridge 2010). Future research on FAB co-cultures could aim to identify the mechanisms mediating the interactions between organisms.

Current methods for treating waterbodies that have been polluted with HMs include neutralization processes, chemical precipitation, coagulation/flocculation, and adsorption; however, these processes suffer from high costs, inefficient HM removal, and may produce secondary pollution (Akcil and Koldas 2006; Hlabela et al 2007; Skousen et al. 2000; Yang et al. 2016). As a result, biotechnological methods are being employed to enhance the bioremediation potential of microbes (Das et al. 2022; Hassan et al. 2017; Higgins et al. 2018; Nazari et al. 2020; Pei et al. 2021; Wang et al. 2021). Several algae have been investigated for bioremediation of textiles, organic pollutants, and HMs (Rehman 2011; Salgueiro et al. 2016; Waluyo et al. 2020; Znad et al. 2018), and recent studies have suggested that co-culturing algae with bacteria or fungi provides benefits that include increased flocculation efficiency, increased biomass, independent nutrient exchange, and enhanced tolerance to extreme environments (Ayangbenro et al. 2019; Fuentes et al. 2016; Lal et al. 2021; Mujtaba et al. 2015; Nazari et al. 2020; Zhang et al. 2021). However, the discoveries here show that *E. mutabilis* and its naturally associated microbial partners offer substantial insight into how organisms interact in a co-culture and provide a model to use for developing enhanced technological applications and potential use in bioremediation.

## Conclusions

This is the first report of treating an *E. mutabilis* culture with antibiotics and a heavy metal. The inclusion of DNA sequencing allowed us to interpret the results, knowing the species present. The results indicated that the culture is a FAB consortium consisting of an HM-tolerant, potentially antibiotic-producing, and plant growth-promoting, fungus *Talaromyces* sp., the extremophilic photobiont *Euglena mutabilis*, and an acidophilic bacterium *Acidiphilium acidophium*. The FAB isolate originated from acid mining runoff in Timmins, Ontario, Canada. Antibiotic and antimycotic suppression of the bacterial and fungal members of the consortium decreased the viability of *E. mutabilis* upon exposure to the CdCl_2_. The indicates these consortium members have a role in responding to Cd stress and possibly other stresses. However, *E. mutabilis* cells that survived had increased chlorophyll production, potentially resulting from Cd supplementing Mg as a component of the chlorophyll pigment. Indicating it can also adapt to the Cd challenge. These interactions, combined with the inability to separate the organisms, suggest interdependence and possibly a tripartite commensal relationship, which we have coined the FAB consortium. This FAB consortium thus can be considered a holobiont consisting of constituent organisms from three separate kingdoms. Together, they can withstand exposure to high concentrations of HM and possibly other stresses. The discovery of this holobiont FAB consortium offers insight into the types of FAB interactions that could be used to create a self-sustaining bioremediation technology.

### Supplementary Information

Below is the link to the electronic supplementary material.

Supplementary file1 (DOCX 4751 KB)

## Data Availability

Raw sequence data obtained from PacBio sequencing are available from the NCBI Sequence Read Archive (https://www.ncbi.nlm.nih.gov/bioproject/PRJNA1026301; accession number PRJNA1026301).
