# Supplementary material for: Euglena mutabilis exists in a FAB consortium with microbes that enhance cadmium tolerance
Source: Int Microbiol. 2024 Jan 3;27(4):1249–68. doi: 10.1007/s10123-023-00474-7 (PMC11300505; doi:10.1007/s10123-023-00474-7)
Supplement: Supplementary file 1 — Supplementary file1 (DOCX 4751 KB) [file 10123_2023_474_MOESM1_ESM.docx]

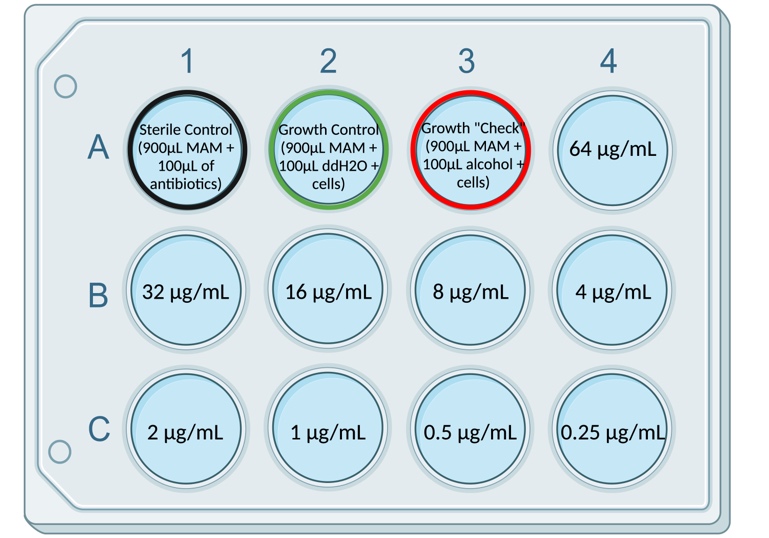

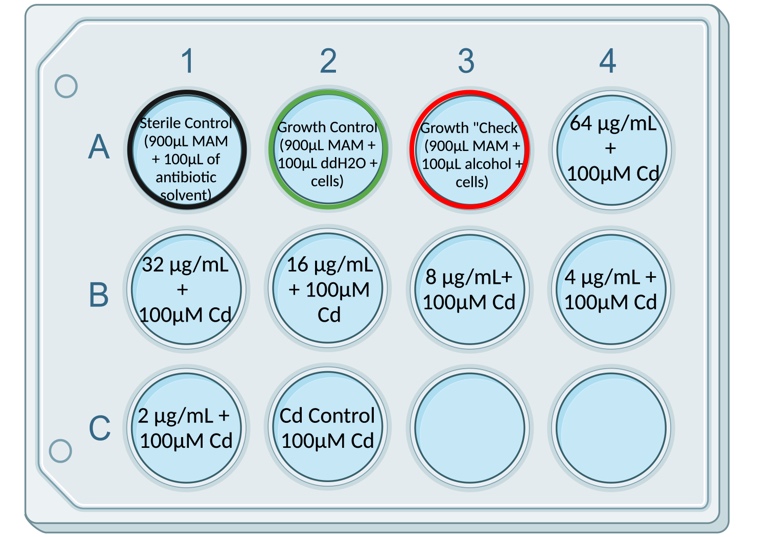

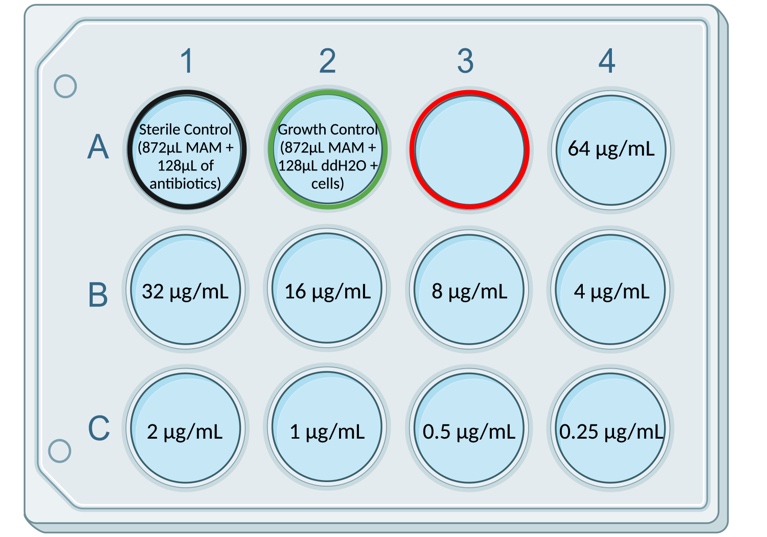

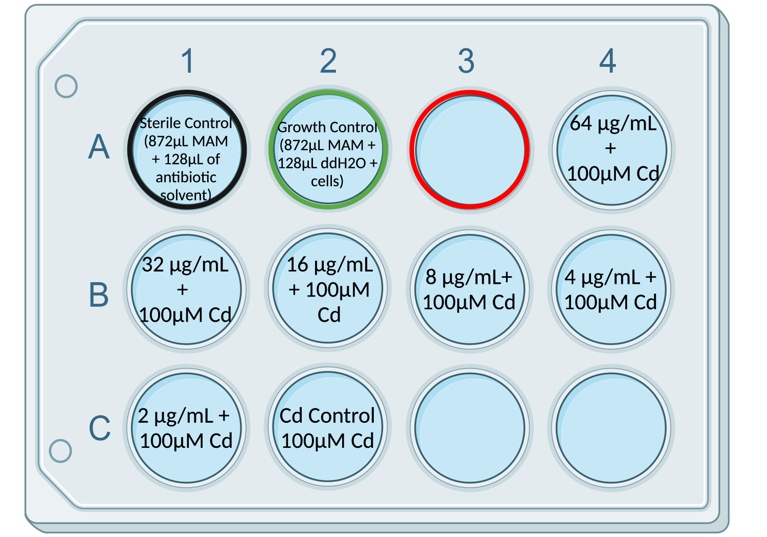
Figure S1: Schematic of a modified minimum inhibitory concentration (MIC) assay in 12-well plates. Each plate contains a sterile control in A1 (MAM + antibiotic) and a growth control in A2 (MAM + water + cells). Antibiotics that have been reconstituted in alcohol (rifampicin, chloramphenicol, cycloheximide) also contain a growth check condition in A3 (MAM + alcohol + cells). The initial treatment of cell cultures with antibiotics begins in A4 with a concentration of 64μg/mL which is serially diluted in subsequent wells to a final concentration of 0.25μg/mL in C4 (a, b). This is repeated with the addition of 100μM CdCl_2_ to assess Cd tolerance against antibiotic treatments ranging from 2-64μg/mL (c, d). Plates containing CdCl_2_ also have a Cd control in C2 (MAM + 100μM CdCl_2_). It can be noted that the lower stock concentration of amphotericin B precludes the addition of the antimycotic at the same volume as other antibiotics in this study, therefore adjustments were made to account for an increase in the volume required to obtain the accurate concentrations required (b, d). Images were created with BioRender.com.

c

d

b

a

Table S1: Relative growth of viable *E. mutabilis* (CPCC 657) and *E. gracilis* (CPCC 95) following 72 hours of antibiotic exposure at various concentrations compared to control conditions (MAM only) where *nil* indicates there was no difference in growth, *ng* indicates no *Euglena* cells were detected, an up arrow (↑) indicates the growth of *Euglena* after antibiotic exposure increase, and a down arrow (↓) indicates the growth of *Euglena* after antibiotic exposure decreased. A star (*) is used to denote the difference in growth (* = p < 0.05, ** = p < 0.01, *** = p < 0.001) determined by a t-test.

| Antibiotic Concentrations (μg/mL) | | *E. mutabilis* | *E. gracilis* |
| --- | --- | --- | --- |
| Kanamycin | 64 | *nil* | *nil* |
|  | 32 | *nil* | *nil* |
|  | 16 | *nil* | *nil* |
|  | 8 | *nil* | *↓ |
|  | 4 | *nil* | *nil* |
|  | 2 | *nil* | *nil* |
| Rifampicin | 64 | *ng* | *ng* |
|  | 32 | ***↓ | ***↓ |
|  | 16 | **↓ | **↓ |
|  | 8 | ***↓ | ***↓ |
|  | 4 | *nil* | **↓ |
|  | 2 | *nil* | ***↓ |
| Chloramphenicol | 64 | *ng* | *ng* |
|  | 32 | *nil* | ***↓ |
|  | 16 | ***↓ | ***↓ |
|  | 8 | ***↓ | ***↓ |
|  | 4 | ***↓ | ***↓ |
|  | 2 | ***↓ | *↓ |
| Tetracycline | 64 | **↓ | *↓ |
|  | 32 | *nil* | *↓ |
|  | 16 | *nil* | **↓ |
|  | 8 | *↓ | **↓ |
|  | 4 | *nil* | **↓ |
|  | 2 | *nil* | **↓ |
| Penicillin Streptomycin | 64 | *nil* | ***↓ |
|  | 32 | *nil* | *↓ |
|  | 16 | *nil* | **↓ |
|  | 8 | *nil* | ***↓ |
|  | 4 | *nil* | *↓ |
|  | 2 | *nil* | **↓ |
| Amphotericin B | 64 | ***↓ | **↓ |
|  | 32 | **↓ | **↓ |
|  | 16 | **↓ | **↓ |
|  | 8 | **↓ | *nil* |
|  | 4 | *nil* | *nil* |
|  | 2 | *nil* | *nil* |

Table S2: ITS and 16S primers selected for the identification of constituent fungal and bacterial organisms in an *E. mutabilis* co-culture (CPCC 657) through Sanger sequencing. ITS primers were selected from Raja et al. (2017), while 16S primers were selected from Thijs et al. (2017).

|  | Primer | Sequence (5’🡪3’) |
| --- | --- | --- |
| ITS | ITS1F | CTTGGTCATTTAGAGGAAGTAA |
|  | ITS1 | TCCGTAGGTGAACCTGCGG |
|  | ITS2 | GCTGCGTTCTTCATCGATGC |
|  | ITS3 | GCATCGATGAAGAACGCAGC |
|  | ITS4 | TCCTCCGCTTATTGATATGC |
|  | ITS5 | GGAAGTAAAAGTCGTAACAAGG |
| 16S | 68F | TNANACATGCAAGTCGRRCG |
|  | 518R | WITACCGCGGCTGCTGG |

Table S3: Cell viability test (*n = 3*) comparing colony forming units of *E. mutabilis* (CPCC 657) and uncharacterized fungal growth, in addition to *E. gracilis* (CPCC 95) after 7 days incubation at 24^o^C in darkness.

|  | *E. mutabilis* | |  | *Fungi* | |  | *E. gracilis* | |
| --- | --- | --- | --- | --- | --- | --- | --- | --- |
|  | *Media Only* | *100 µM CdCl_2_* |  | *Media Only* | *100 µM CdCl_2_* |  | *Media Only* | *100 µM CdCl_2_* |
| Control | +++ | ++ |  | - | +++ |  | ++ | +++ |
|  | *16 μg/mL* | *16 µg/mL + 100 µM CdCl_2_* |  | *16 μg/mL* | *16 μg/mL + 100 µM CdCl_2_* |  | *16 μg/mL* | *16 µg/mL + 100 µM CdCl_2_* |
| Kanamycin | + | + |  | - | +++ |  | ++ | +++ |
| Rifampicin | +++ | + |  | + | +++ |  | + | + |
| Chloramphenicol | ++ | + |  | - | + |  | + | ++ |
| Tetracycline | ++ | + |  | + | +++ |  | + | ++ |
| Penicillin-Streptomycin | ++ | + |  | - | +++ |  | + | ++ |
| Amphotericin B | + | + |  | + | +++ |  | + | +++ |
|  |  |  |  |  |  |  |  |  |
|  | *8 μg/mL* | *8 µg/mL + 100 µM CdCl_2_* |  | *8 μg/mL* | *8 μg/mL + 100 µM CdCl_2_* |  | *8 μg/mL* | *8 µg/mL + 100 µM CdCl_2_* |
| Kanamycin | + | + |  | - | +++ |  | ++ | +++ |
| Rifampicin | ++ | + |  | + | +++ |  | + | + |
| Chloramphenicol | ++ | + |  | + | + |  | + | ++ |
| Tetracycline | +++ | + |  | + | +++ |  | + | ++ |
| Penicillin-Streptomycin | + | + |  | - | +++ |  | + | +++ |
| Amphotericin B | + | + |  | - | +++ |  | + | + |
|  |  |  |  |  |  |  |  |  |
|  | *4 μg/mL* | *4 µg/mL + 100 µM CdCl_2_* |  | *4 μg/mL* | *4 μg/mL + 100 µM CdCl_2_* |  | *4 μg/mL* | *4 µg/mL + 100 µM CdCl_2_* |
| Kanamycin | + | + |  | - | +++ |  | ++ | +++ |
| Rifampicin | ++ | ++ |  | + | +++ |  | + | + |
| Chloramphenicol | +++ | +++ |  | + | + |  | + | ++ |
| Tetracycline | ++ | ++ |  | + | +++ |  | + | ++ |
| Penicillin-Streptomycin | + | + |  | - | +++ |  | + | ++ |
| Amphotericin B | + | + |  | + | +++ |  | + | ++ |
|  |  |  |  |  |  |  |  |  |
|  | *2 μg/mL* | *2 µg/mL + 100 µM CdCl_2_* |  | *2 μg/mL* | *2 μg/mL + 100 µM CdCl_2_* |  | *2 μg/mL* | *2 µg/mL + 100 µM CdCl_2_* |
| Kanamycin | + | + |  | - | +++ |  | ++ | +++ |
| Rifampicin | ++ | + |  | + | +++ |  | + | + |
| Chloramphenicol | ++ | + |  | + | +++ |  | + | +++ |
| Tetracycline | +++ | + |  | - | +++ |  | + | ++ |
| Penicillin-Streptomycin | + | + |  | + | +++ |  | + | ++ |
| Amphotericin B | + | + |  | + | +++ |  | + | ++ |

*(–) no CFU, (+) if < 50 CFU, (+ +) if 50 ><150 CFU, and (+ + +) if >150 or complete lawn present and CFU count impossible.*


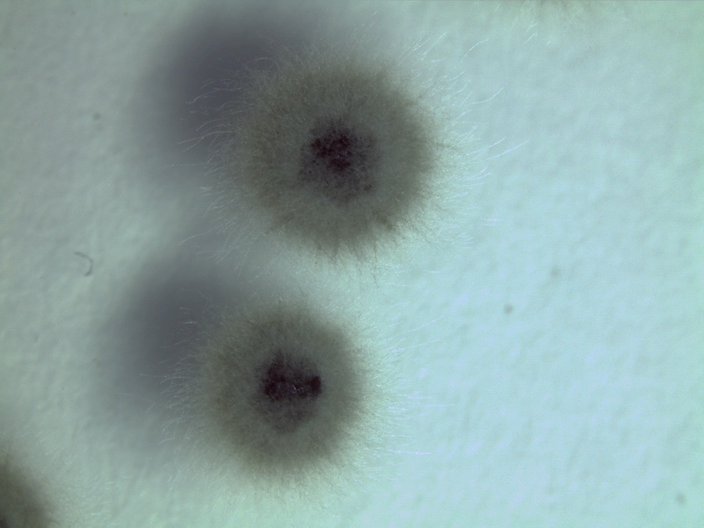

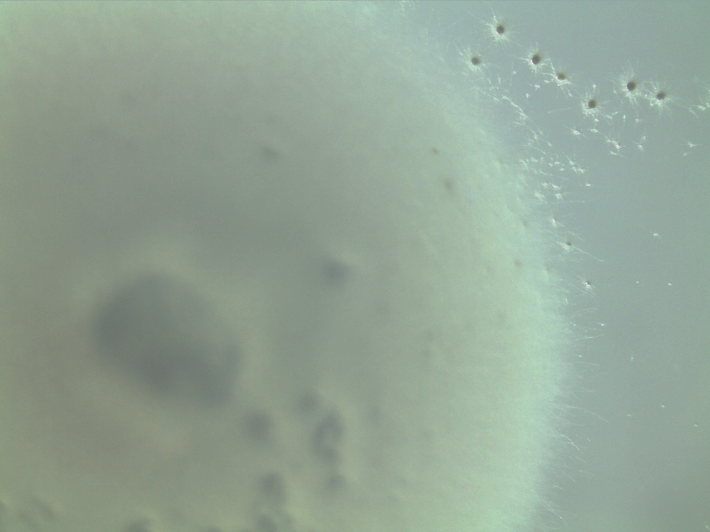


b

a


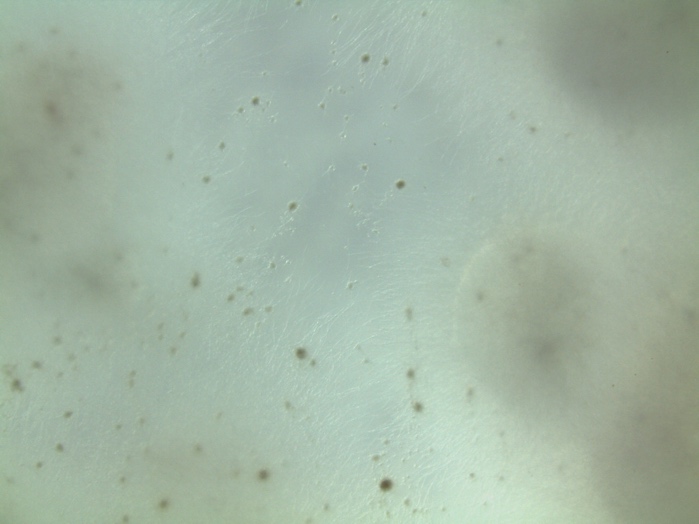

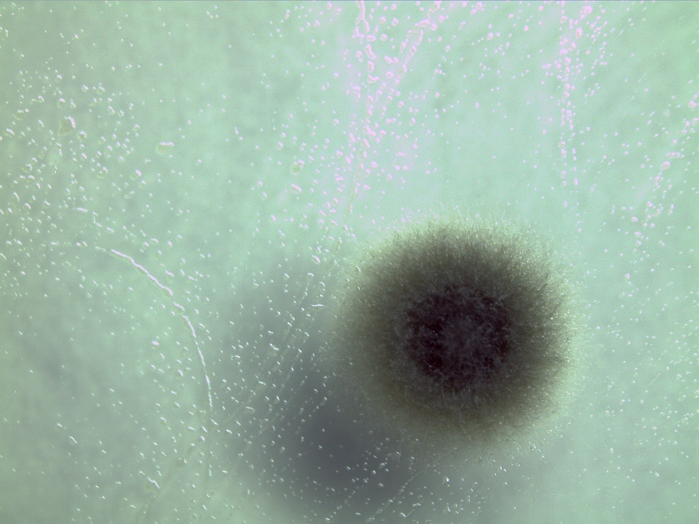

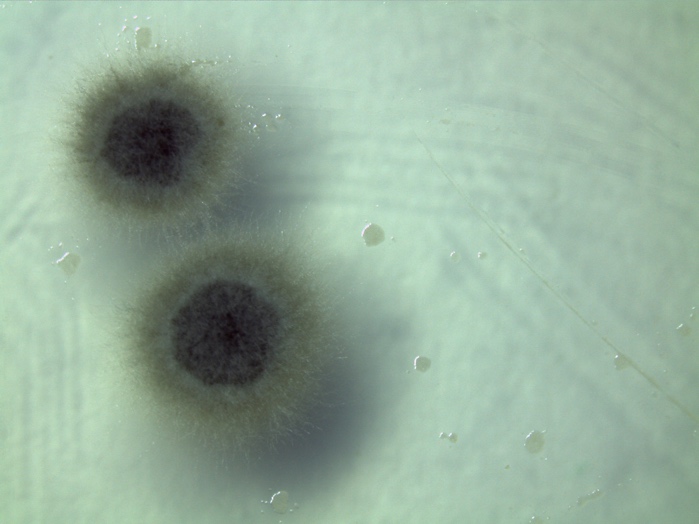


e

d

c

Figure S2: Plate photographs of an *E. mutabilis* co-culture (CPCC657) after 7 days on R2A plates in control conditions (a), following 72 hours of exposure to 8 µg/mL of cycloheximide (b), 64 µg/mL of cycloheximide (c), 32 µg/mL of kanamycin (d), and after 7 days on PDA plates following 72 hours of exposure to 4 µ/mL of amphotericin B and 100 µM CdCl_2_ (e). Growth of *E. mutabilis* exhibits intimate interactions with constituent bacterial and fungal organisms which prohibits the isolation on single colonies.

a

b

c

Figure S3: Plate photographs of an *E. mutabilis* co-culture (CPCC 657) after 7 days on PDA plates following 72 hours of exposure to 64μg/mL of cycloheximide (a) and 2μg/mL of exposure to amphotericin B (b, c).
